# IL-6 gene *rs1800795* polymorphism and diabetes mellitus: a comprehensive analysis involving 42,150 participants from a meta-analysis

**DOI:** 10.1186/s13098-022-00851-8

**Published:** 2022-07-15

**Authors:** Zhiying Cheng, Chunmin Zhang, Yuanyuan Mi

**Affiliations:** 1 General Practice, DeltaHealth Hospital, Shanghai, China; 2 Xinqiao Town Community Health Service Center, Songjiang District, Shanghai, China; 3 Department of Urology, Affiliated Hospital of Jiangnan University, Wuxi, China

**Keywords:** Interleukin-6, Type 2 diabetes mellitus, Type 1 diabetes mellitus, Polymorphism, Risk, Meta-analysis

## Abstract

**Background:**

Over the past two decades, several studies have focused on the association between a common polymorphism (*rs1800795*) from interleukin-6 (IL-6) gene and Diabetes Mellitus (DM) risk. However, the results remain ambiguous and indefinite.

**Methods:**

A comprehensive analysis was performed to explore this relationship. A search was conducted in the PubMed, Embase, Chinese (CNKI and Wanfang), and GWAS Catalog databases, covering all publications until February 10, 2022. Odds ratios (OR) with 95% confidence intervals (CI) were used to evaluate the strength of the association. Publication bias was assessed using both Begg and Egger tests.

**Results:**

Overall, 34 case–control studies with 7257 T2DM patients and 15,598 controls, and 12 case–control studies (10,264 T1DM patients and 9031 health controls) were included in the analysis. A significantly lower association was observed between the *rs1800795* polymorphism and T2DM risk in Asians, mixed population, and hospital-based (HB) subgroups (C-allele vs. G-allele: OR = 0.76, 95% CI  0.58–0.99, *P* = 0.039 for Asians; CG vs. GG: OR = 0.74, 95% CI  0.58–0.94, *P* = 0.014 for mixed population; CC vs. GG: OR = 0.61, 95% CI  0.41–0.90, *P* = 0.014 for HB). However, increased associations were found from total, mixed population, and HB subgroups between *rs1800795* polymorphism and T1DM susceptibility (CG vs. GG: OR = 1.32, 95% CI 1.01–1.74, *P* = 0.043 for total population, CC vs. GG: OR = 2.45, 95% CI 1.18–5.07, *P* = 0.016 for mixed individuals; C-allele vs. G-allele: OR = 1.29, 95% CI 1.07–1.56, *P* = 0.0009 for HB subgroup).

**Conclusions:**

In summary, there is definite evidence to confirm that *IL-6 rs1800795* polymorphism is associated with susceptibility to decreased T2DM and increased T1DM.

**Supplementary Information:**

The online version contains supplementary material available at 10.1186/s13098-022-00851-8.

## Background

Diabetes mellitus (DM) is a chronic medical condition in which the body either produces too little insulin from pancreatic islets or lacks effective access to insulin [1]. Type 1 DM (T1DM) is most often diagnosed in children and adolescents with respect to islet function development. Type 2 DM (T2DM) is caused by insulin resistance, and the body cannot use insulin effectively and may gradually lose its production capacity [2–4]. To the best of our knowledge, age, obesity, and family history are the major risk factors of developing DM [5]. However, the exact pathogenesis of DM is not fully understood. Past genome-wide association studies (GWAS) have identified over 100 genetic sites, which suggests that there are significant associations between different sites and susceptibility to DM, indicating that genetic factors may be crucial for its occurrence and development [6, 7].

Interleukin-6 (IL-6), a classic proinflammatory cytokine, plays a prominent role in the inflammatory response and is associated with insulin resistance and T2DM [8]. In addition, chronic low-grade inflammation and activation of the innate immune system are closely associated with the pathogenesis of T1DM and its complications. Inflammatory cytokines such as IL-6 are determinants of these pathogenic processes [9, 10].

The IL-6 gene is located on chromosome 7p21. The gene, which includes seven exons, covers approximately 12.8 kb of genomic DNA [11]. A common single nucleotide polymorphism (SNP) in the IL-6 promoter in T2DM has been named *rs1800795* (also named –174G/C) [12]. The *rs1800795* polymorphism functionally affects IL-6 promoter activity, indicating that the carried CC genotype individual is associated with lower plasma levels of IL-6 compared with individuals with the GG genotype [13]. In addition, the G-allele in homozygotes (GG genotype) was associated with higher concentrations of IL-6, increasing the immune response [14, 15], demonstrating that this polymorphism is functional, or that it defined a difference in IL-6 expression levels according to the genotype of the polymorphism.

Several epidemiological studies have observed associations between genetic variants of IL-6 and the risk of DM. For instance, Saxena et al. observed that the *rs1800795* polymorphism showed a highly significant association with T2DM [16]. In contrast, Dhamodharan et al. determined that the C allele conferred significant protection against T2DM [17]. In addition, Fathy et al. [18] demonstrated a lack of significant association between *rs1800795* polymorphism and T2DM. For T1DM, an increased association was observed between T1DM and the polymorphism by Cooper et al. [19]. However, Tsiavou et al. observed no significant differences [20]. Two meta-analyses (Yin and Xu et al.) showed that *rs1800795* is not associated with T1DM risk [21, 22]. On the other hand, Huth and Xia et al. performed a meta-analysis and concluded that this polymorphism could be associated with a decreased risk of T2DM [23, 24]. In the last 10 years, some larger and more comprehensive studies have been conducted on this association. Therefore, it is necessary to perform an updated meta-analysis to understand the associations between *rs1800795* polymorphism and T1DM/T2DM [12, 15–20, 25–60].

## Materials and methods

### Document retrieval and data extraction

We used online databases, including PubMed, Embase, CNKI, Wanfang, and GWAS Catalog (https://www.ebi.ac.uk/gwas/) until on Feb 10, 2022, with keywords including ‘Interleukin-6/IL-6’, ‘polymorphism/variant’, and ‘Diabetes Mellitus/DM/TIDM/T2DM’. Two researchers (Zhiying Cheng, Chunmin Zhang) evaluated the articles to identify the stages through the abstract and then the full article. Systematic analysis/meta-analysis, case studies, other polymorphisms, insufficient data for each genotype, and duplications were identified and removed from further analysis. In addition, our meta-analysis was performed according to the Preferred Reporting Items for Systematic Reviews and Meta-Analyses (PRISMA) (Additional file 1: Table S1) and Meta-analysis of Observational Studies in Epidemiology. This study was registered at PROSPERO (number 329822; https://www.crd.york.ac.uk/prospero/). Eligible studies were selected based on the following criteria: @) studies assessing the association between TIDM or T2DMAdditional file: As per journal requirements, every additional file must have a corresponding caption. In this regard, please be informed that the caption was taken from the Additional file 1 itself. Please advise if action taken appropriate and amend if necessary. and *rs1800795* variants; @) case/control studies; and @) age-and sex-matched control subjects. The exclusion criteria were: @) not case/control studies; @) insufficient genotype frequency; @) duplicate studies; and @) significantly biased articles. Information including the name of the first author, year of publication, origin, race, DM type, genotype methods, and Hardy–Weinberg equilibrium (HWE) was collected.

### Quality assessment

Quality was assessed using the Newcastle–Ottawa Scale (NOS) for cross-sectional study quality assessment. The methodological quality of each study (sampling strategy, response rate, and representativeness), comparability, and outcomes were assessed using the NOS tool. Studies with a score of more than 7 out of 10 were considered suitable. This cutoff point was determined after reviewing relevant meta-analyses from the literature [61–63].

### Statistical analyses

The correlation between *IL-6 rs1800795* polymorphism and the risk of TIDM/T2DM was measured using 95% confidence interval (CI) and odds-ratio (OR) according to the genotype frequencies of the case and control groups. Ethnic groups were divided into African, mixed, Caucasian, and Asian groups. Population-based (PB) and hospital-based (HB) control subgroups were also identified.

The statistical significance of the results was calculated using the *Z*-test. In these studies, the heterogeneity hypothesis was assessed using the *Q*-test based on the chi-squared test [64]. If significant heterogeneity (< 0.1) was detected, the random effects model was used, else the fixed effects model was selected [65, 66]. For *IL-6 rs1800795*, we studied the relationship between variation and the risk of T2DM in the C-allele vs. G-allele, CG vs. GG, and CC + CG vs. GG models; and C-allele vs. G-allele, CC vs. GG, CC vs. CG + GG, CG vs. GG, and CC + CG vs. GG models for T1DM risk. The asymmetry of the funnel plot was evaluated using Begg’s test, and publication bias was evaluated using Egger’s test. Statistical significance was set at *P* < 0.05 [67]. Pearson’s chi-squared test was used in the control group (*P* < 0.05), and the *χ*^2^ test was used to evaluate the deviation of *rs1800795* polymorphism from the expected frequency of HWE [68]. All statistical tests were conducted using Stata (version 11.0; StataCorp LP, College Station, Texas, USA). The power of our meta-analysis was calculated online using the website http://www.power-analysis.com/.

### Gene interaction network analysis of the IL-6 gene

To fully understand the role of IL-6 and its potential functional partners in DM, we used the STRING online server (http://string-db.org/) to construct an IL-6 gene–gene interaction network.

## Results

### Study selection and characteristics

**Fig. 1:**
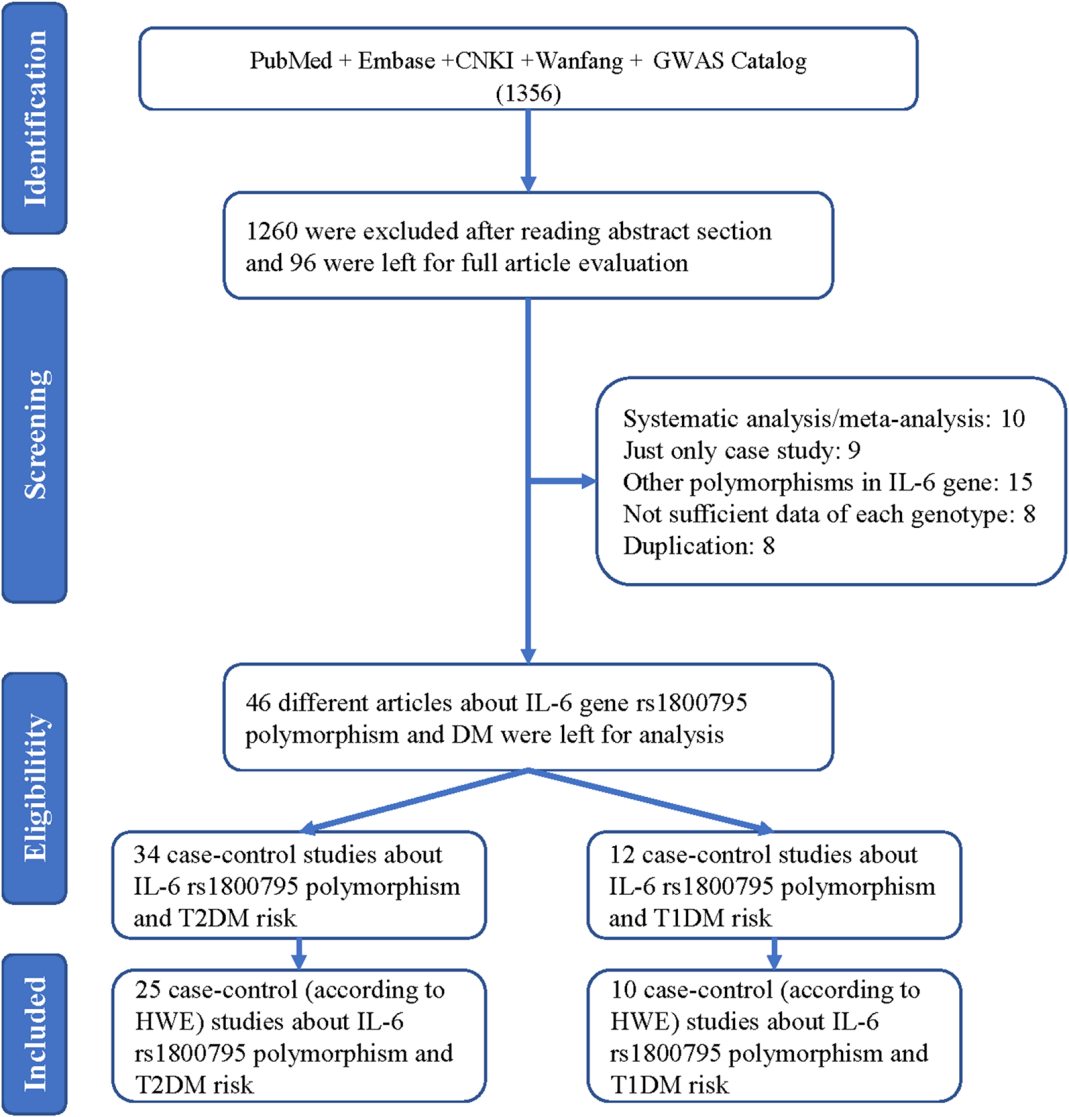
A flowchart illustrating the search strategy used to identify association studies for *IL-6 rs1800795* polymorphism and DM risk

**Fig. 2:**
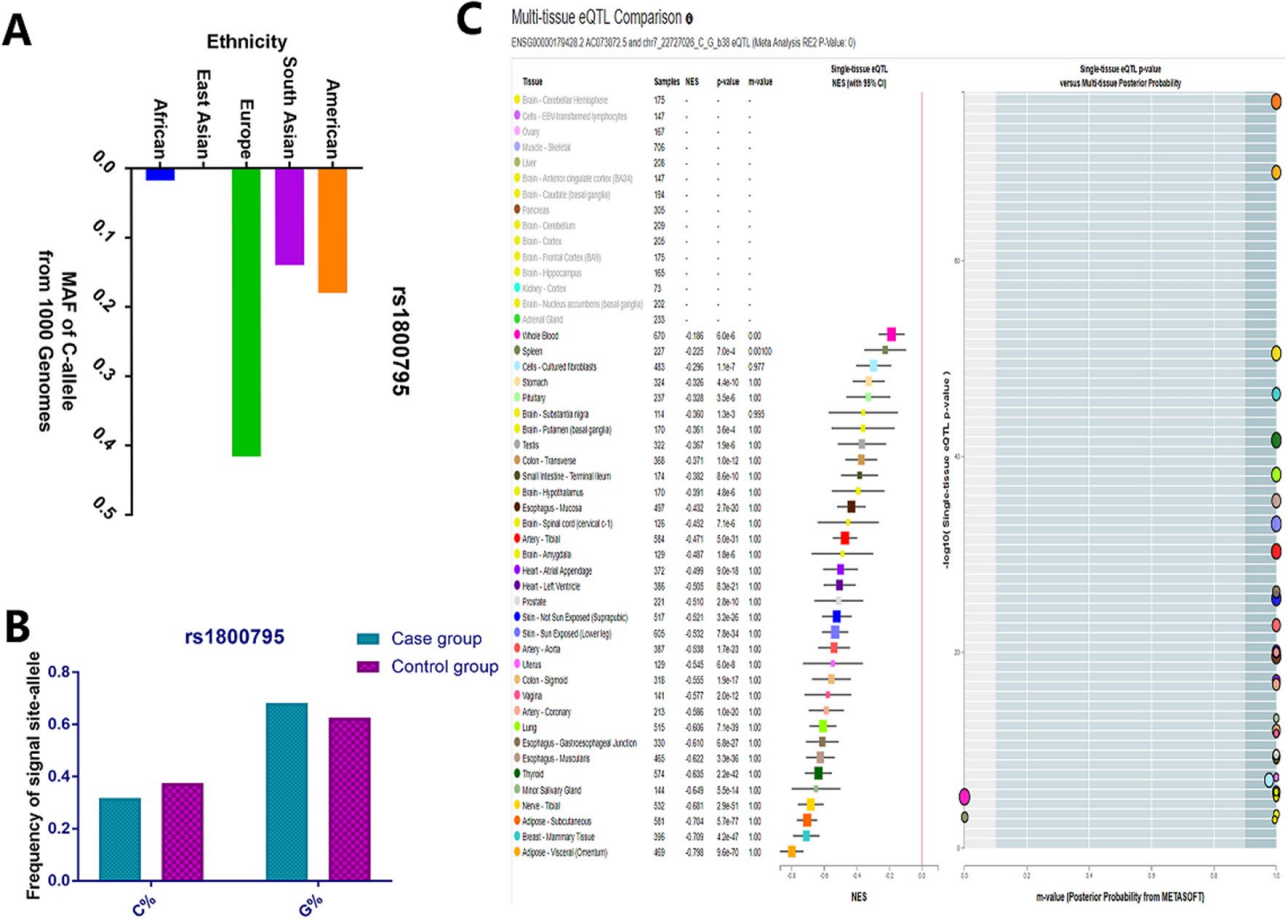
**A** The MAF of minor-allele (mutant-allele) for *IL-6 rs1800795* polymorphism from the 1000 Genomes online database. **B** The frequency about C-allele or G-allele both in case and control groups. **C** The risk frequency of rs*1800795* polymorphism to several disease from TCGA database

A total of 1356 articles were identified from the four main databases (PubMed, Embase, CNKI, and Wanfang). 1260 papers were excluded after reading the abstract, and 96 articles were used for a complete evaluation. Among them, 50 articles were excluded for the following reasons: systematic analysis/meta-analysis (10), only case studies (9), other polymorphisms in the IL-6 gene (15), insufficient data for each genotype (8), and duplication (8) (Fig. 1). Thus, 46 papers [13–18] accounting for a total of 17,521 DM patients and 24,629 healthy controls were included in our meta-analysis (34 case–control studies including 7257 T2DM patients and 15,598 controls, and 12 case–control studies including 17,521 T1DM and 9031 controls) [12, 15–20, 25–60] (Table 1). We checked the minor allele frequency (MAF) reported for the five main populations worldwide in the 1000 Genomes Browser (https://www.ncbi.nlm.nih.gov/snp/rs1800795#frequency_tab) (Fig. 2A). In addition, the C-allele frequency was significantly lower in both cases and controls (Fig. 2B) (Table 2). The relationship between this polymorphism and several organs is shown in Fig. 2C (https://www.gtexportal.org/home/). The distribution of genotypes in controls was not consistent with the HWE in T2DM (9 case–control studies) [15, 26, 32, 38, 41, 42, 51, 53, 60] and T1DM (2 case–control studies) [44, 48] (Table 1). Genotyping of the SNPs of IL-6 gene *rs1800795* polymorphism was conducted using the genotyping methods listed in Table 1.

**Table 1:** Characteristics of studies of IL-6 rs1800795 polymorphism and T2DM and T1DM risk included in our meta-analysis

Author	Year	Country	Ethnicity	Type	Case	Control	SOC	Cases			Controls			HWE	Genotype	NOS
								CC	CG	GG	CC	CG	GG			
Campos	2019	Brasil	Mixed	T1DM	141	150	HB	9	69	63	7	68	75	0.084	PCR–RFLP	6
Mysliwiec	2008	Poland	Caucasian	T1DM	200	172	HB	59	105	36	43	75	54	0.103	PCR–RFLP	8
Siekiera	2002	Poland	Caucasian	T1DM	36	36	HB	5	24	7	12	18	6	0.684	PCR-SSP	6
Ururahy	2015	Brazil	Mixed	T1DM	120	152	HB	9	41	70	4	45	103	0.727	TaqMan	8
Settin	2009	Egypt	African	T1DM	50	98	PB	9	38	3	6	87	5	< 0.05	PCR-SSP	7
Javor	2010	Slovakia	Caucasian	T1DM	151	140	PB	31	85	35	21	66	53	0.951	PCR-SSP	7
Cooper	2007	USA	Caucasian	T1DM	8852	7785	PB	1612	4312	2928	1515	3814	2456	0.619	Taqman	7
Jahromi	2000	England	Caucasian	T1DM	257	120	PB	32	95	130	29	51	40	0.118	sequence	7
Tsiavou	2004	Greece	Caucasian	T1DM	31	39	PB	3	11	17	3	11	25	0.281	PCR-SSP	8
Mysliwska	2009	Poland	Caucasian	T1DM	210	170	PB	69	110	31	51	68	51	< 0.05	PCR–RFLP	8
Perez-Bravo	2011	Chile	Mixed	T1DM	145	103	PB	6	49	90	1	27	75	0.396	PCR–RFLP	7
Mukhopadhyaya	2010	India	Asian	T2DM	40	40	PB	6	11	23	15	13	12	0.029	PCR–RFLP	8
Hamid	2005	Denmark	Caucasian	T2DM	1389	4401	PB	328	659	402	1022	2133	1246	0.062	MALDI-TOF	9
Plataki	2018	Greece	Caucasian	T2DM	144	180	HB	12	64	68	12	54	114	0.119	PCR–RFLP	6
Vozarova	2003	Spain	Caucasian	T2DM	211	118	PB	17	110	84	19	65	34	0.193	PCR–RFLP	8
Buraczynska	2016	Poland	Caucasian	T2DM	1090	612	PB	240	534	316	129	288	195	0.237	sequence	9
Chen	2002	China	Asian	T2DM	196	130	HB	40	84	72	42	58	30	0.254	PCR–RFLP	7
Tsiavou	2004	Greece	Caucasian	T2DM	31	39	HB	3	11	17	3	11	25	0.281	PCR–SSP	6
Eze	2016	Switzerland	Caucasian	T2DM	286	5560	HB	40	135	111	865	2614	2081	0.352	Taqman	7
Bouhaha	2010	Tunisia	African	T2DM	169	281	PB	4	40	125	7	64	210	0.428	Sequencing	8
Ghavimi	2016	Iran	Asian	T2DM	120	120	HB	18	62	40	27	64	29	0.463	PCR–RFLP	7
Fathy	2018	Kuwait	Asian	T2DM	50	42	HB	1	13	36	2	11	29	0.487	TaqMan	8
Lara-Gómez	2019	Mexico	Mixed	T2DM	31	30	HB	1	11	19	0	5	25	0.618	Sequencing	7
Dhamodharan	2015	India	Asian	T2DM	139	106	HB	1	46	92	12	44	50	0.626	PCR–RFLP	7
Danielsson	2005	Sweden	Caucasian	T2DM	20	20	HB	6	12	2	6	9	5	0.662	Sequencing	7
Vozarova	2003	Spain	Caucasian	T2DM	143	145	PB	0	1	142	0	9	136	0.699	PCR–RFLP	9
Neelofar	2017	India	Asian	T2DM	50	50	HB	3	19	28	3	20	27	0.78	sequence	7
Kavitha	2016	India	Asian	T2DM	30	30	HB	0	0	30	0	1	29	0.926	PCR–RFLP	6
Kong	2010	China	Asian	T2DM	107	121	HB	0	2	105	0	2	119	0.927	PCR-SSP	6
Zhang	2011	China	Asian	T2DM	512	483	HB	0	2	510	0	1	482	0.982	PCR–RFLP	7
Saxena	2014	India	Asian	T2DM	213	145	HB	4	46	163	19	21	105	< 0.05	PCR–RFLP	6
Xiao	2009	China	Asian	T2DM	85	132	HB	0	0	85	0	0	132	< 0.05	PCR–RFLP	7
Nadeem	2017	Pakistan	Asian	T2DM	539	250	HB	37	267	235	48	74	128	< 0.05	PCR–RFLP	6
Karadeniz	2014	Turkey	Caucasian	T2DM	86	340	HB	6	27	53	26	171	143	< 0.05	PCR–RFLP	6
Erdogan	2017	Turkey	Caucasian	T2DM	35	119	HB	1	16	18	16	79	24	< 0.05	PCR–RFLP	8
Helaly	2013	Egypt	African	T2DM	69	98	PB	18	49	2	6	87	5	< 0.05	an allele–specific PCR	8
Mohlig	2004	Germany	Caucasian	T2DM	188	376	PB	32	103	53	71	208	97	< 0.05	SNuPE	8

*HB* hospital-based; *PB* population-based; *SOC* source of control; *PCR–RFLP* polymerase chain reaction followed by restriction fragment length polymorphism; *PCR-SSP* polymerase chain reaction followed with sequence specific primers; *MALDI-TOF* a chip-based matrix-assisted laser-desorption/ionization time-of-flight; *HWE* Hardy–Weinberg equilibrium of control group; *NOS* Newcastle–Ottawa Scale

**Table 2:** The Minor Allele Frequency (MAF) reported for the five main worldwide populations in the 1000 Genomes Browser and the C-allele or G-allele frequency both in cases and controls of this study

Study	Population	Group	Sample size	Ref. allele (C)	Alt. allele (G)
1000Genomes	African	Sub	1322	0.0182	0.9818
1000Genomes	East Asian	Sub	1008	0.0010	0.9990
1000Genomes	Europen	Sub	1006	0.4155	0.5845
1000Genomes	South Asian	Sub	978	0.139	0.861
1000Genomes	American	Sub	694	0.184	0.816
Current study	Total	Case	17,520	0.3817	0.6183
Current study	Total	Control	24,629	0.394	0.606

### IL-6 *rs1800795* polymorphism and T2DM risk

**Fig. 3:**
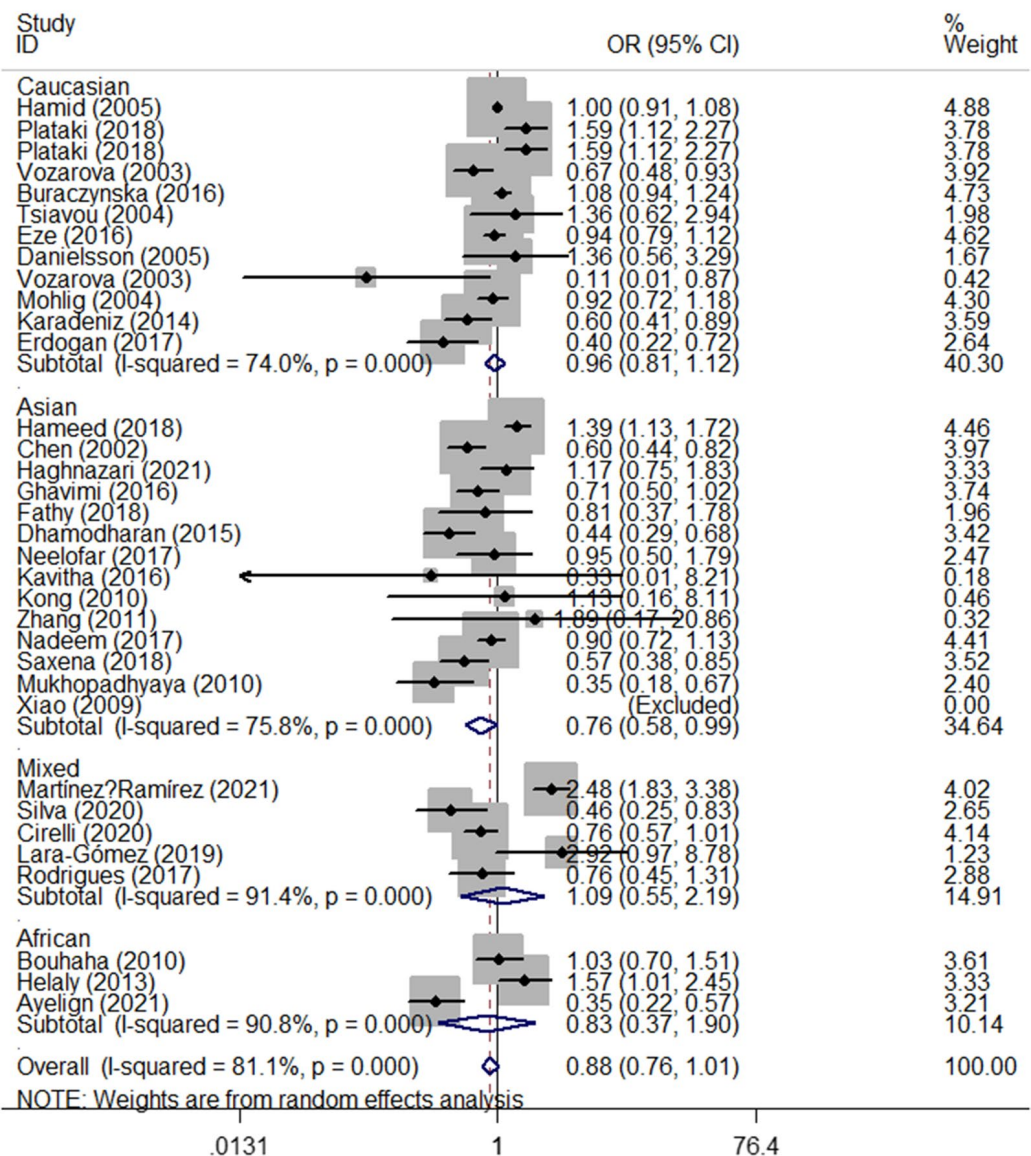
Forest plot of T2DM risk associated with *IL-6 rs1800795* polymorphism (C-allele vs. G-allele) in the subgroup of Asian subgroup. The squares and hori*z*ontal lines correspond to the study-specific OR and 95% CI. The area of the squares reflects the weight (inverse of the variance). The diamond represents the summary OR and 95% CI

**Fig. 4:**
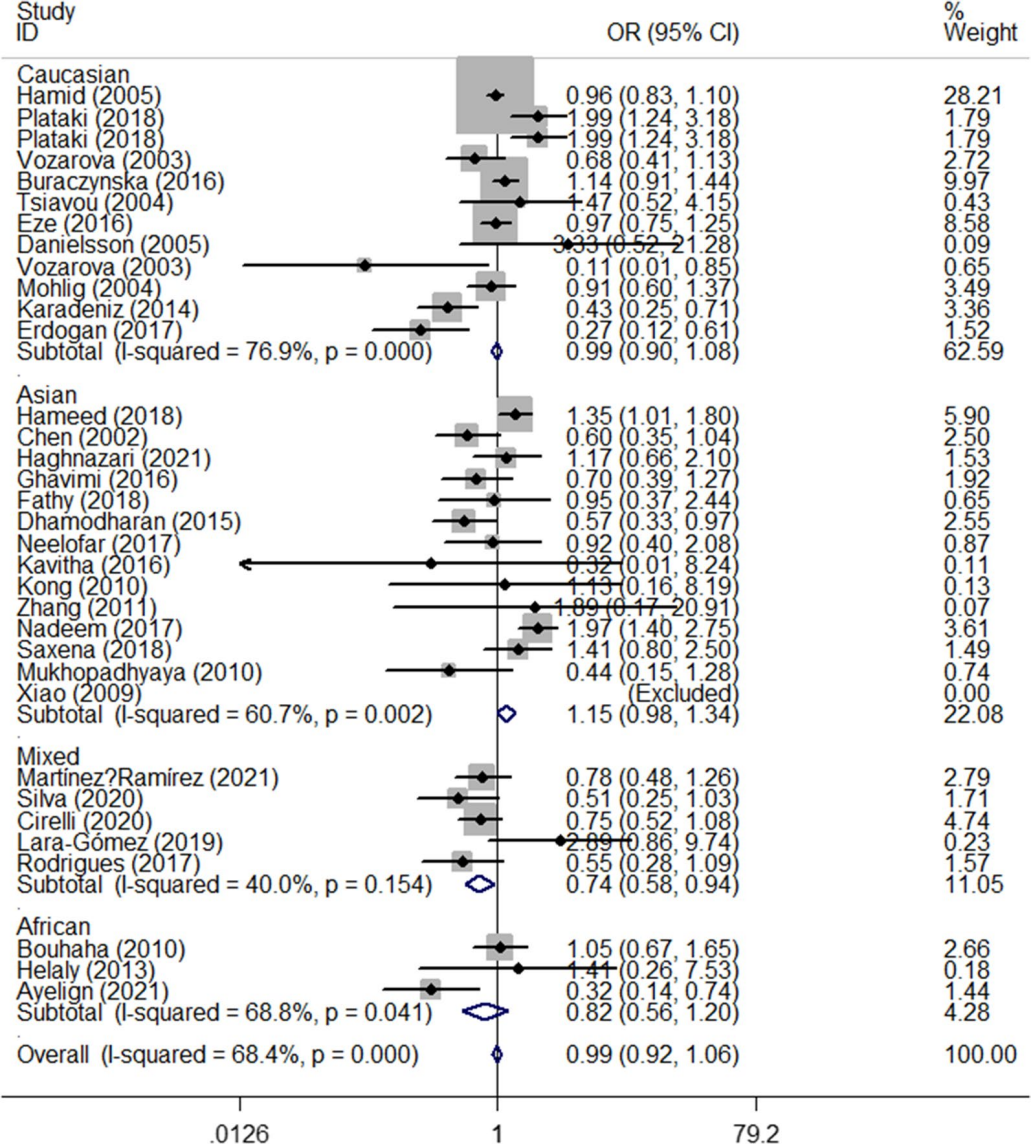
Forest plot of T2DM risk associated with *IL-6 rs1800795* polymorphism (CG vs. GG) in the subgroup of Mixed subgroup

**Fig. 5:**
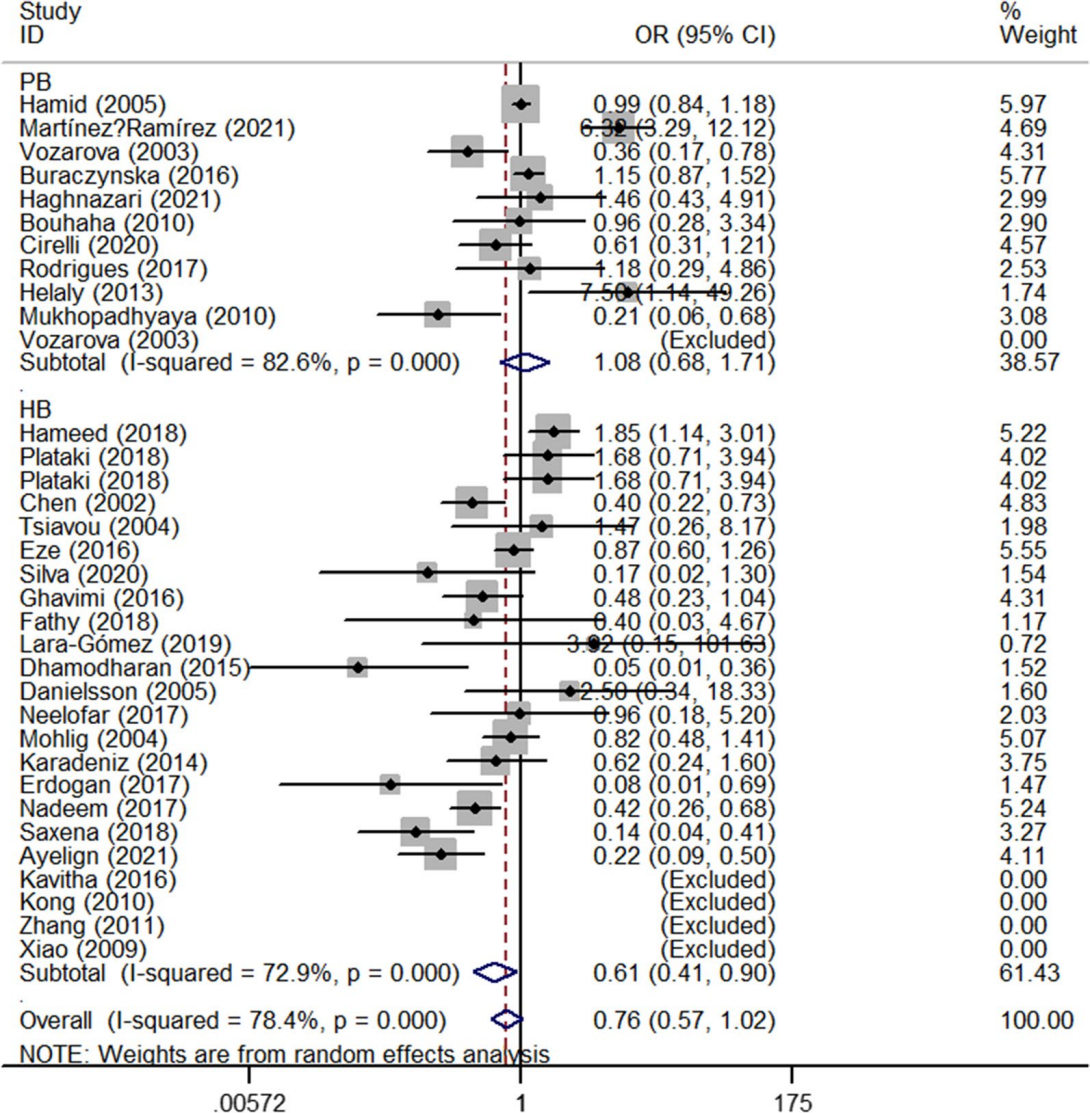
Forest plot of T2DM risk associated with *IL-6 rs1800795* polymorphism (CC vs. GG) in the subgroup of HB subgroup

The results of the meta-analysis suggested no associations between IL-6 rs1800795 polymorphism and T2DM risk (Table 3). If studies that were not consistent with HWE were excluded, no significant results were detected in any of the three models. Analysis of ethnicity subgroups showed a statistically significant association in Asians (OR_C-allele vs. G-allele_ = 0.76, 95% CI 0.58–0.99, *P* = 0.039, random effect model; OR_CC vs. GG_ = 0.45, 95% CI 0.24–0.85, *P* = 0.014, random effect model, OR_CC vs. CG+GG_ = 0.48, 95% CI 0.27–0.86, *P* = 0.014, random effect model, Fig. 3) and mixed populations (OR_CG vs. GG_ = 0.74, 95% CI 0.58–0.94, *P* = 0.014, fixed effect model**, **Fig. 4). Surprisingly, a marginal and poorly significant difference was found in the HB sources of the control subgroup (OR_CC vs. GG_ = 0.61, 95% CI 0.41–0.90, *P* < 0.011, random effect model, OR_CC vs. CG+GG_ = 0.64, 95% CI 0.46–0.90, *P* = 0.011, random effect model, Fig. 5). Furthermore, if studies that were not consistent with HWE were included, no significant association was found between Asians and HB subgroups (Table 3).

**Table 3:** Stratified analyses of IL-6 rs1800795 polymorphism and T2DM and T1DM risk

Variables	N0.	Case/	C-allele vs. G-allele			CG vs. GG		
		Control	OR(95%CI)	Ph	P	OR (95% CI)	Ph	P
T2DM								
Total	34	7257/15598	0.88 (0.76–1.01)	0.000	0.075	0.91 (0.77–1.08)	0.000	0.281
HWE	25	5927/14023	0.98 (0.84–1.15)	0.000	0.832	0.97 (0.83–1.13)	0.000	0.687
Ethnicity								
Asian	14	2595/2208	0.76 (0.58–0.99)	0.000	0.039	0.99 (0.73–1.32)	0.002	0.925
Caucasian	12	3767/12090	0.96 (0.81–1.12)	0.000	0.579	0.95 (0.73–1.22)	0.000	0.683
Mixed	5	582/846	1.09 (0.55–2.19)	0.000	0.804	0.74 (0.58–0.94)	0.154	0.014
African	3	313/454	0.83 (0.37–1.89)	0.000	0.665	0.91 (0.77–1.08)	0.041	0.486
SOC								
HB	23	3546/9186	0.83 (0.68–1.01)	0.000	0.059	0.95 (0.73–1.23)	0.000	0.706
PB	11	3711/6412	0.98 (0.79–1.22)	0.000	0.874	0.89 (0.75–1.05)	0.100	0.166
Ethnicity (with HWE)								
Asian	10	1887/1922	0.83 (0.59–1.17)	0.000	0.283	0.97 (0.81–1.17)	0.115	0.778
Caucasian	9	3458/11255	1.06 (0.91–1.25)	0.001	0.443	1.15 (0.90–1.47)	0.001	0.257
Mixed	5	582/846	1.09 (0.55–2.19)	0.000	0.804	0.74 (0.58–0.94)	0.154	0.014
SOC (with HWE)								
HB	16	2325/7749	0.97 (0.76–1.24)	0.000	0.788	1.07 (0.82–1.39)	0.001	0.635
PB	9	3602/6274	1.01 (0.81–1.26)	0.000	0.907	0.90 (0.76–1.07)	0.086	0.221
T1DM								
Total	12	10,264/9031	1.17 (0.96–1.42)	0.000	0.120	1.32 (1.01–1.74)	0.000	0.043
HWE	10	10,004/8763	1.13 (0.91–1.41)	0.000	0.268	1.24 (0.96–1.61)	0.002	0.100
Ethnicity								
Caucasian	7	9737/8462	1.06 (0.81–1.38)	0.000	0.682	1.37 (0.90–2.11)	0.000	0.146
Mixed	3	406/405	1.39 (1.10–1.77)	0.497	0.006	1.33 (0.99–1.79)	0.835	0.059
SOC								
HB	4	497/510	1.29 (1.07–1.56)	0.122	0.009	1.47 (1.11–1.94)	0.428	0.008
PB	8	9767/8521	1.15 (0.89–1.48)	0.000	0.276	1.27 (0.88–1.82)	0.000	0.195
Ethnicity (with HWE)								
Caucasian	6	9527/8292	0.99 (0.74–1.34)	0.000	0.971	1.21 (0.80–1.82)	0.001	0.368
Mixed	3	406/405	1.39 (1.10–1.77)	0.497	0.006	1.33 (0.99–1.79)	0.835	0.059
SOC (with HWE)								
HB	4	497/510	1.29 (1.07–1.56)	0.122	0.009	1.47 (1.11–1.94)	0.428	0.008
PB	6	9507/8253	1.09 (0.80–1.49)	0.000	0.578	1.13 (0.81–1.58)	0.010	0.460

Variables	CC + CG vs. GG			CC vs. GG			CC vs. CG + GG		
	OR (95% CI)	Ph	P	OR (95% CI)	Ph	P	OR (95% CI)	Ph	P
T2DM									
Total	0.87 (0.73–1.03)	0.000	0.039	0.76 (0.57–1.02)	0.000	0.039	0.82 (0.63–1.07)	0.000	0.039
HWE	0.98 (0.82–1.16)	0.000	0.786	0.98 (0.72–1.33)	0.000	0.905	0.99 (0.76–1.29)	0.000	0.962
Ethnicity									
Asian	0.82 (0.61–1.11)	0.000	0.208	0.45 (0.24–0.85)	0.000	0.014	0.48 (0.27–0.86)	0.000	0.014
Caucasian	0.93 (0.72–1.20)	0.000	0.569	0.94 (0.74–1.19)	0.043	0.595	0.98 (0.89–1.10)	0.513	0.778
Mixed	0.94 (0.53–1.67)	0.000	0.833	1.25 (0.30–5.19)	0.000	0.759	1.38 (0.35–5.54)	0.000	0.645
African	0.71 (0.23–2.13)	0.003	0.536	0.99 (0.15–6.34)	0.002	0.991	1.22 (0.22–6.67)	0.000	0.818
SOC									
HB	0.85 (0.66–1.10)	0.000	0.227	0.61 (0.41–0.90)	0.000	0.014	0.64 (0.46–0.90)	0.000	0.011
PB	0.92 (0.73–1.14)	0.001	0.430	1.08 (0.68–1.71)	0.000	0.751	1.21 (0.79–1.85)	0.000	0.373
Ethnicity (with HWE)									
Asian	0.82 (0.56–1.20)	0.002	0.303	0.62 (0.28–1.38)	0.000	0.241	0.72 (0.38–1.35)	0.004	0.305
Caucasian	1.13 (0.89–1.44)	0.000	0.316	1.01 (0.80–1.28)	0.098	0.918	1.00 (0.90–1.12)	0.495	0.979
Mixed	0.94 (0.53–1.67)	0.000	0.833	1.25 (0.30–5.19)	0.000	0.759	1.38 (0.35–5.54)	0.000	0.645
SOC (with HWE)									
HB	1.01 (0.75–1.37)	0.000	0.932	0.85 (0.53–1.36)	0.000	0.493	0.85 (0.60–1.21)	0.026	0.369
PB	0.96 (0.77–1.18)	0.003	0.677	1.13 (0.72–1.79)	0.000	0.590	0.99 (0.78–1.80)	0.000	0.426
T1DM									
Total	1.32 (0.99–1.76)	0.000	0.060	1.40 (0.90–2.18)	0.000	0.134	1.12 (0.83–1.50)	0.005	0.463
HWE	1.25 (0.94–1.67)	0.000	0.131	1.27 (0.78–2.05)	0.000	0.331	1.04 (0.74–1.45)	0.014	0.839
Ethnicity									
Caucasian	1.30 (0.83–2.01)	0.000	0.249	1.31 (0.67–1.92)	0.000	0.645	0.93 (0.69–1.23)	0.024	0.598
Mixed	1.43 (1.07–1.90)	0.724	0.015	2.45 (1.18–5.07)	0.486	0.016	2.20 (1.08–4.48)	0.487	0.031
SOC									
HB	1.51 (1.15–1.98)	0.337	0.003	1.77 (1.14–2.74)	0.128	0.010	1.16 (0.57–2.35)	0.066	
PB	1.27 (0.86–1.86)	0.000	0.229	1.33 (0.77–2.32)	0.000	0.312	1.10 (0.77–1.57)	0.013	0.611
Ethnicity (with HWE)									
Caucasian	1.15 (0.74–1.78)	0.00	0.540	0.99 (0.56–1.75)	0.000	0.968	0.88 (0.61–1.25)	0.018	0.465
Mixed	1.43 (1.07–1.90)	0.724	0.015	2.45 (1.18–5.07)	0.486	0.016	2.20 (1.08–4.48)	0.487	0.031
SOC (with HWE)									
HB	1.51 (1.15–1.98)	0.337	0.003	1.77 (1.14–2.74)	0.128	0.010	1.16 (0.57–2.35)	0.066	0.680
PB	1.14 (0.78–1.68)	0.000	0.494	1.11 (0.59–2.10)	0.001	0.746	0.97 (0.62–1.50)	0.041	0.887

*P*_*h*_ value of *Q*-test for heterogeneity test; *P* *Z*-test for the statistical significance of the OR; *SOC* source of control, *HB* hospital-based, *PB* population-based

### IL-6 *rs1800795* polymorphism and T1DM risk.

**Fig. 6:**
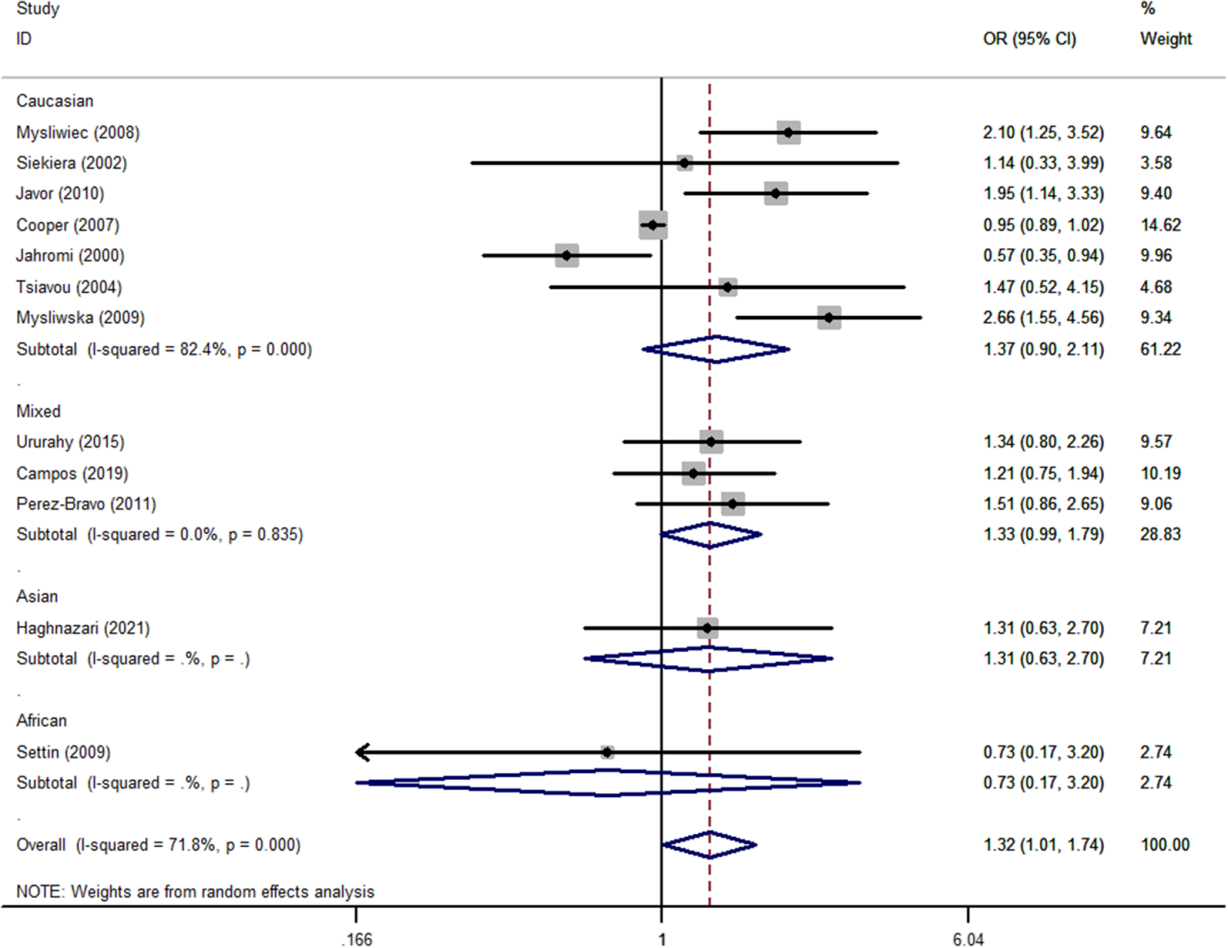
Forest plot of T1DM risk associated with *IL-6 rs1800795* polymorphism (CG vs. GG) in the whole

**Fig. 7:**
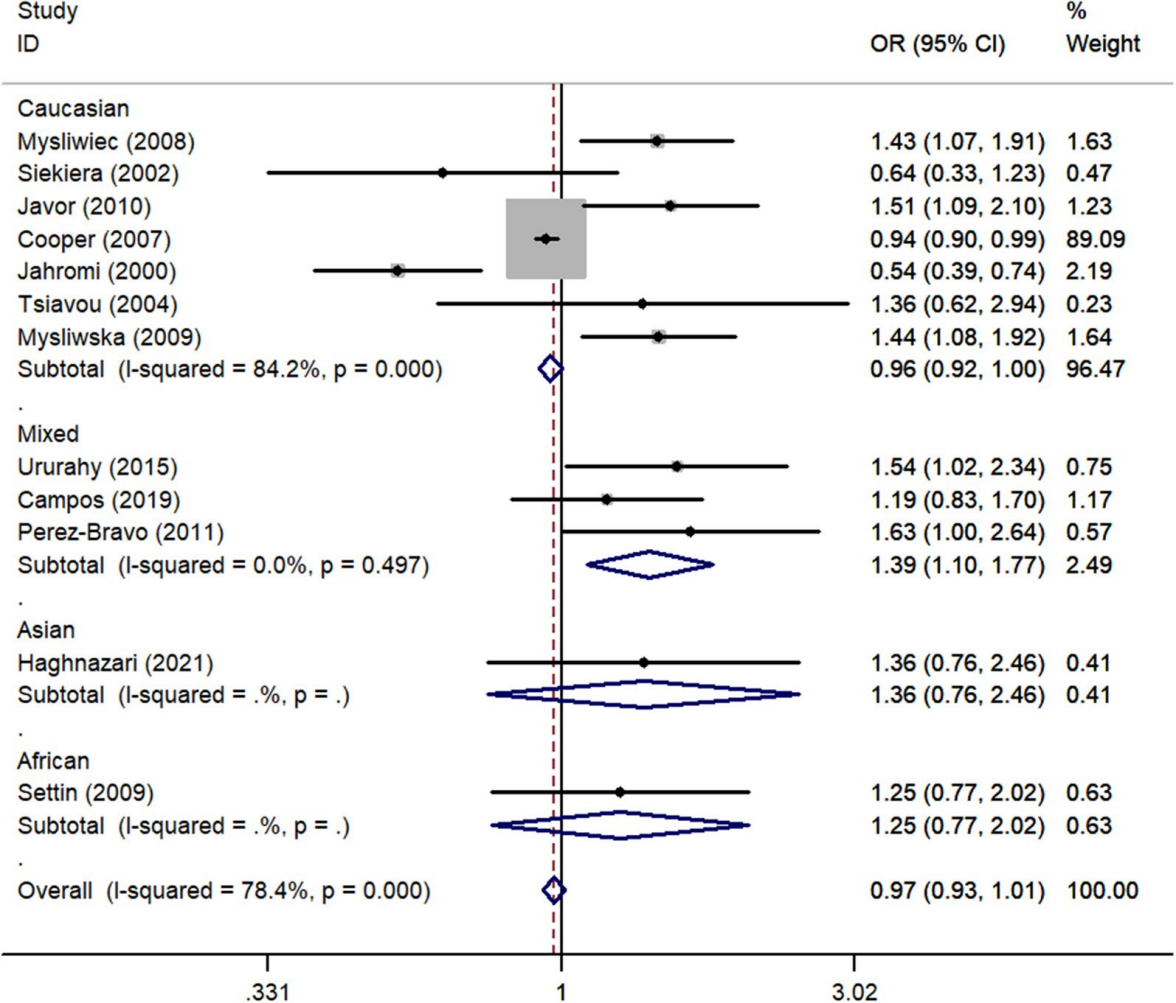
Forest plot of T1DM risk associated with *IL-6 rs1800795* polymorphism (C-allele vs. G-allele) in the Mixed subgroup

**Fig. 8:**
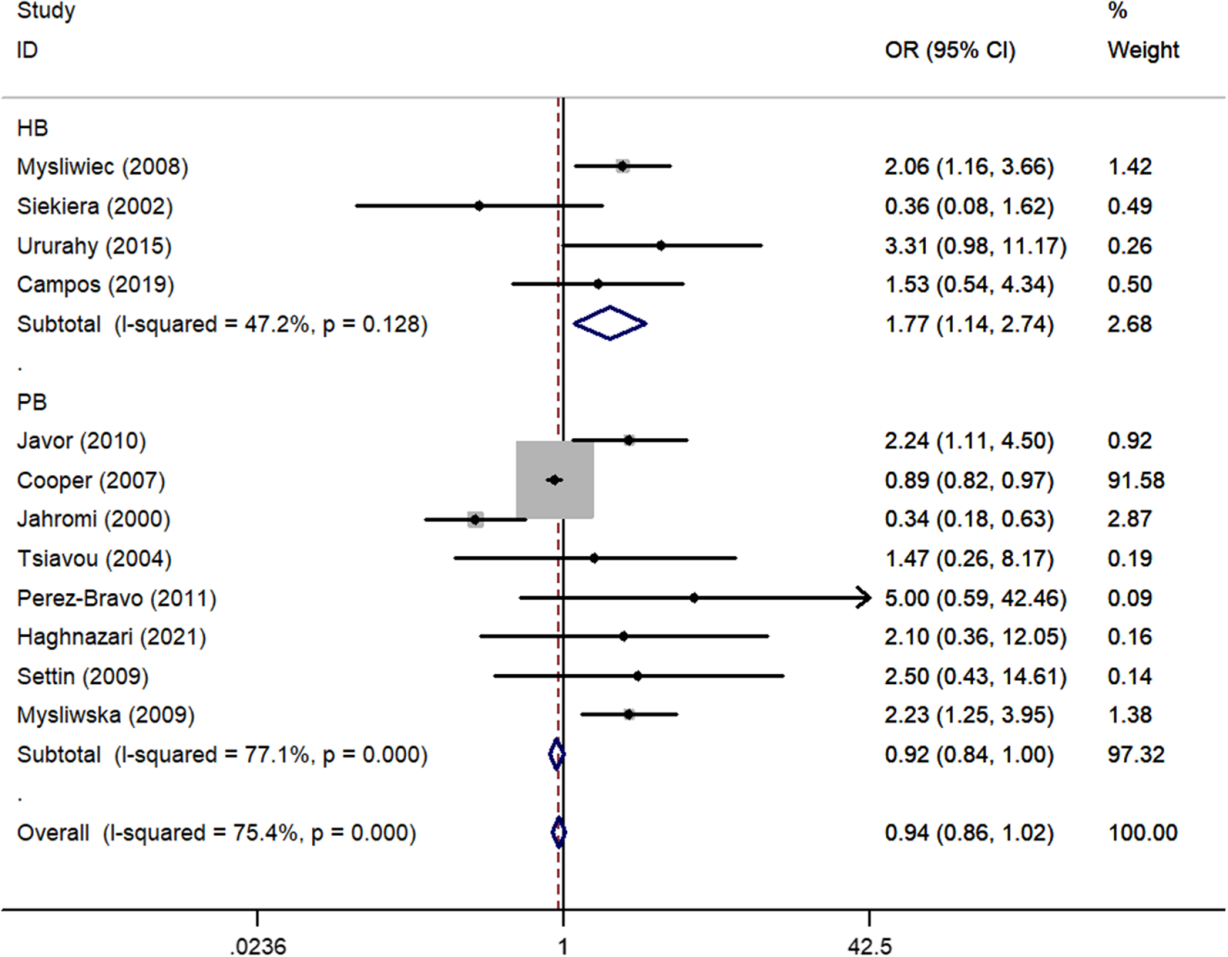
Forest plot of T1DM risk associated with *IL-6 rs1800795* polymorphism (CC vs. GG) in the HB subgroup

There was a significant positive association between *rs1800795* polymorphism and T1DM susceptibility in the total analysis (OR_CC vs. GG_ = 1.32, 95% CI 1.01–1.74, *P* = 0.043, random effect model, Fig. 6) (Table 3). Additionally, a risk association was observed between this polymorphism in the mixed population (OR_C-allele vs. G-allele_ = 1.39, 95% CI 1.10–1.77, *P* = 0.006, fixed effect model, OR_CC vs. GG_ = 2.45, 95% CI 1.18–5.07, *P* = 0.016, fixed effect model, OR_CC+CG vs. GG_ = 1.43, 95% CI 1.07–1.90, *P* = 0.015, fixed effect model, OR_CC vs. CG+GG_ = 2.20, 95% CI 1.08–4.48, *P* = 0.031, fixed effect model, Fig. 7). Similar relationships were observed for the sources of the HB subgroup (OR_C-allele vs. G-allele_ = 1.29, 95% CI 1.07–1.56, *P* = 0.009, fixed effect model, OR_CG vs. GG_ = 1.47, 95% CI 1.11–1.94, *P* = 0.008, fixed effect model, Fig. 8). Furthermore, when we excluded studies that were not consistent with HWE, the results remain the same as above (Table 3).

### Publication bias and sensitive analysis

**Fig. 9:**
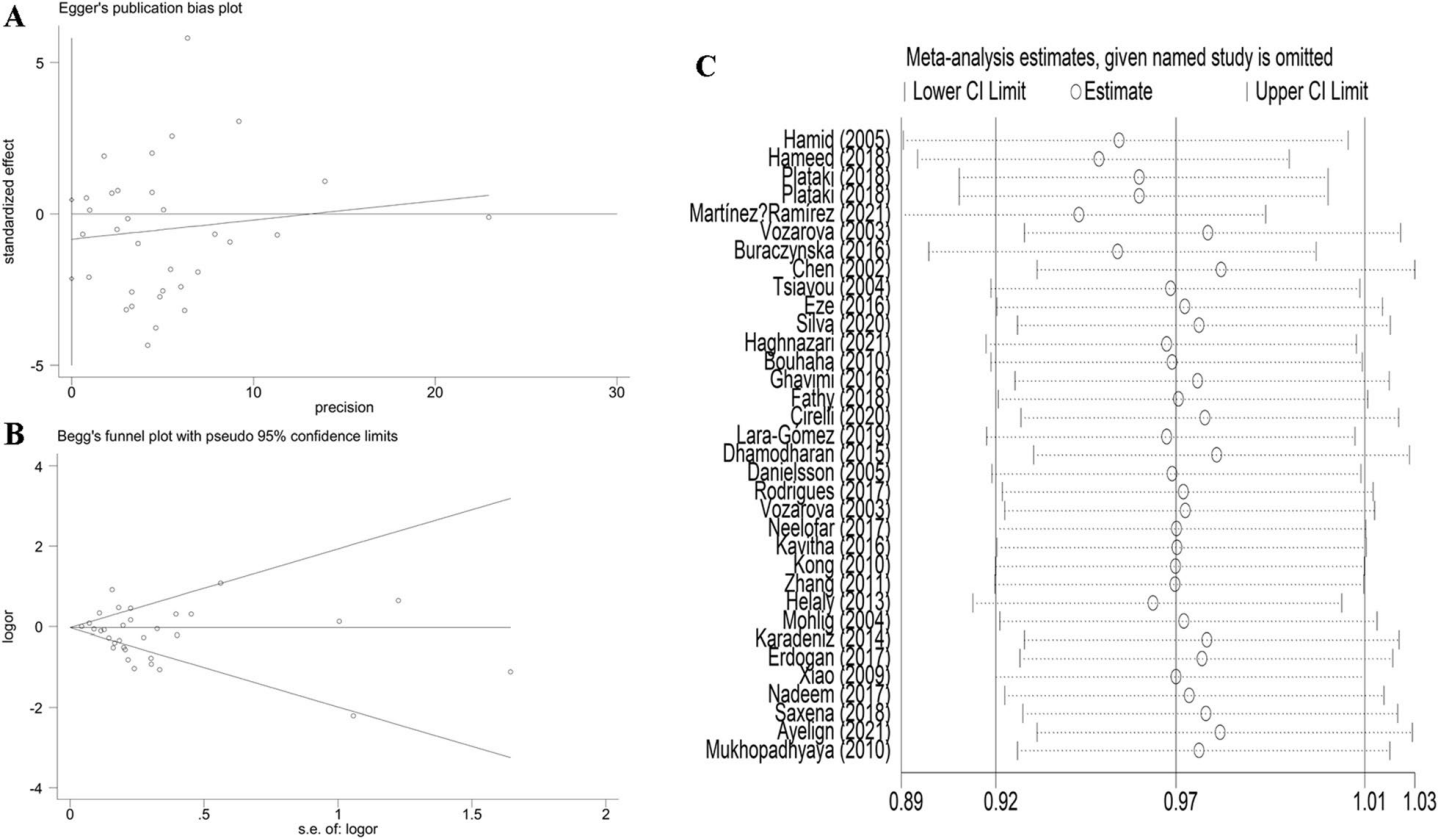
A: Begg’s funnel plot for publication bias test (C-allele vs. G-allele). B: Egger’s publication bias plot (C-allele vs. G-allele) for T2DM. Sensitivity analysis between *IL-6 rs1800795* polymorphism and T2DM risk (C-allele vs. G-allele)

**Fig. 10:**
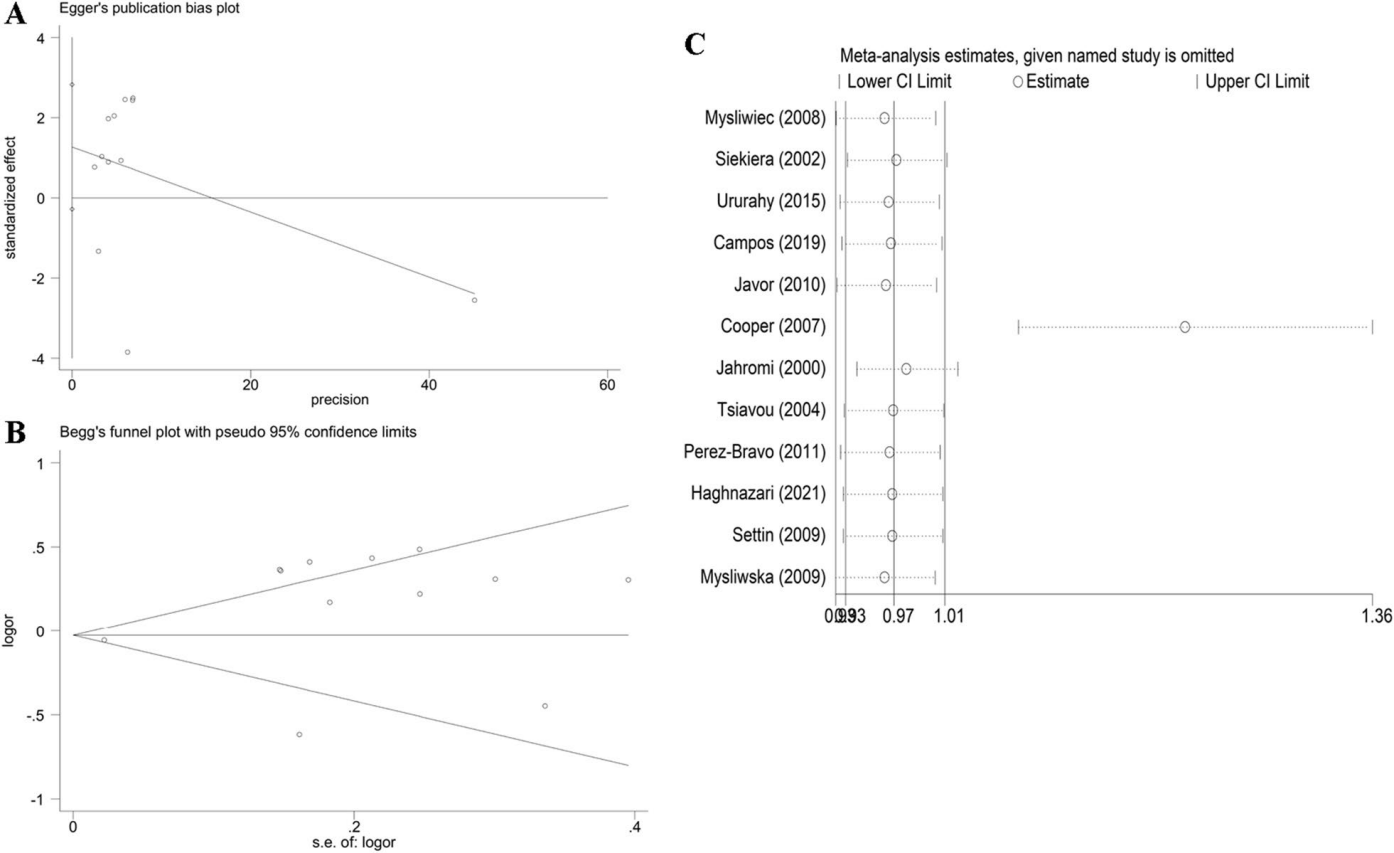
**A** Begg’s funnel plot for publication bias test (C-allele vs. G-allele). **B** Egger’s publication bias plot (C-allele vs. G-allele) for T1DM. Sensitivity analysis between *IL-6 rs1800795* polymorphism and T1DM risk (C-allele vs. G-allele)

Begg’s and Egger’s tests were performed to assess publication bias, which was not found for T2DM or T1DM analyses (T2DM: *t*_C-allele vs. G-allele_ =  − 1.32, *P* = 0.195 for Egger’s test, *z* = 1.02, *P* = 0.306 for Begg’s test, Fig. 9a, b; T1DM: *t*_C-allele vs. G-allele_ = 1.82, *P* = 0.099 for Egger’s test, *z* = 1.17, *P* = 0.244 for Begg’s test, Fig. 10a,b, Table 4). To delete studies that may influence the power and stability of the whole study, we applied a sensitivity analysis, and no sensitive case–control studies were found (Figs. 9c, 10c, Table 4).

**Table 4:** Publication bias tests (Begg’s funnel plot and Egger’s test for publication bias test) for IL-6 rs1800795 polymorphism and T2DM and T1DM risk

Egger's test						Begg's test	
Genetic type	Coefficient	Standard error	t	P value	95%CI of intercept	z	P value
T2DM							
C-allele vs. G-allele	− 0.842	0.636	− 1.32	0.195	(− 2.139–0.455)	1.02	0.306
CG vs. GG	− 0.688	0.469	− 1.47	0.152	(− 1.645–0.268)	1.18	0.239
CC + CG vs. GG	− 0.756	0.511	− 1.48	0.149	(− 1.799–0.287)	1.05	0.292
CC vs. GG	− 0.318	0.301	− 1.06	0.301	(− 0.636–0.300)	0.34	0.736
CC vs. CG + GG	− 0.304	0.32	− 0.95	0.351	(− 0.961–0.353)	0.45	0.653
T1DM							
C-allele vs. G-allele	1.268	0.697	1.82	0.099	(− 0.286–2.823)	1.17	0.244
CG vs. GG	0.858	0.454	1.89	0.088	(− 0.152–1.869)	− 0.07	1
CC + CG vs. GG	0.894	0.481	1.86	0.093	(− 0.178–1.967)	0.21	0.837
CC vs. GG	0.455	0.323	1.41	0.189	(− 0.265–1.174)	0.75	0.451
CC vs. CG + GG	0.523	0.384	1.36	0.202	(− 0.331–1.379)	0.34	0.732

### Gene–gene network diagram and interactions

**Fig. 11:**
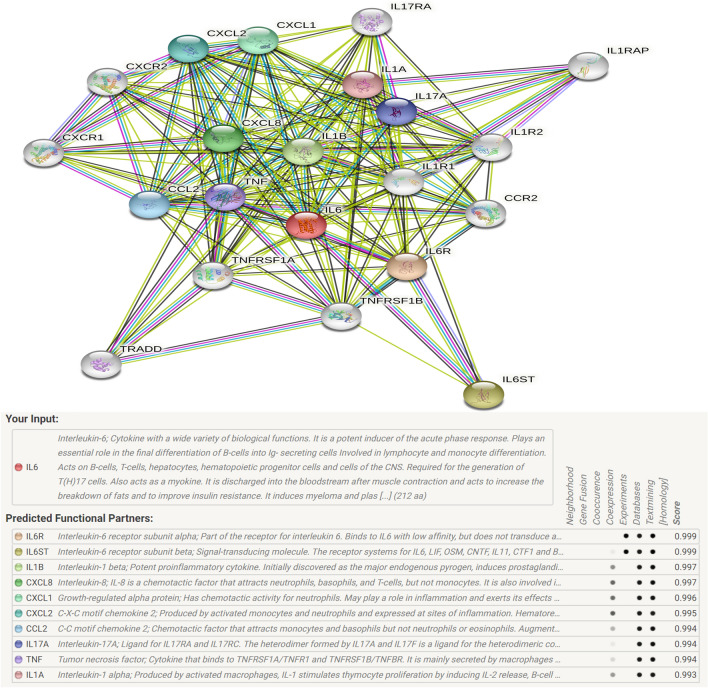
Human IL-6 interactions network with other genes obtained from String server. At least 10 genes have been indicated to correlate with IL-6 gene. **A** the gene–gene interaction; **B** the detail of relative ten core genes

Our analysis using the STRING online server indicated that IL-6 interacts with several genes. The ten most significant genes from the network of gene–gene interactions are shown in Fig. 11. These ten genes are: interleukin-6 receptor (IL6R); interleukin-6 receptor subunit beta (IL6ST); interleukin-1 beta (IL1B); interleukin-8 (CXCL8); growth-regulated alpha protein (CXCL1); C-X-C motif chemokine 2 (CXCL2); C–C motif chemokine 2 (CCL2); interleukin-17A (IL17A); tumor necrosis factor (TNF); and interleukin-1 alpha (IL1A).

## Discussion

Diabetes has reached pandemic dimensions, and is becoming relevant in both developed and developing countries, affecting over 400 million people worldwide [69]. To date, several studies have focused on the relationship between *IL-6 rs1800795* polymorphism and DM risk [26, 29, 30, 38]. A few meta-analysis-based studies have also indicated similar associations [21–24]. However, there is a lack of robust conclusions. Therefore, it is necessary to recombine previously published studies to perform a comprehensive meta-analysis to understand the above-mentioned association in further detail. To the best of our knowledge, meta-analysis is a powerful method when the results are based on a large number of samples and are inconsistent, including different ethnicities or countries [24]. The conclusion obtained from the meta-analysis is more robust than that of a single study [24]. To investigate the association between *IL-6 rs1800795* and DM, our comprehensive study included 42,150 individuals. Our results indicate that *IL-6 rs1800795* acts as a protective factor in T2DM. In other words, individuals carrying the C-allele may have a decreased association with T2DM, particularly among Asians, mixed populations, and HB source studies. However, *IL-6 rs1800795* was found to be a risk factor for T1DM, and there was a significantly increased association between this polymorphism and T1DM risk in four genetic models in mixed-population and HB source studies.

Therefore, *IL-6 rs1800795* polymorphism may have different effects in different types of DM, and also have different influences on different ethnicities, such as Asians and mixed populations. This could be due to the following: the pathogenic mechanisms of T2DM and T1DM are different, with differences in several significantly expressed genes. Further studies should focus on the functions and mechanisms of mutation or wild-type *IL-6 rs1800795* polymorphism to define the dissimilarity between T2DM and T1DM. On the other hand, the same gene may have different effects, even opposite, and the IL-6 gene may behave differently for T2DM and T1DM. Therefore, *rs1800795* polymorphism affecting the expression of IL-6 may also differ in its roles in T2DM and T1DM. Different races have heterogeneity, and the same gene may also have different roles in different ethnicities [70, 71]. Third, heterogeneity in the selection strategy may exist, which may have affected our results. To evaluate the stability and validity of the current study, we performed a power analysis. The power in T2DM was 1 and that in T1DM was 0.166, indicating that the conclusions from T2DM were more powerful and persuasive than those for T1DM. This suggests that more studies on *rs1800795* and T1DM risk should be conducted in future to obtain a robust conclusion.

The development and outcome of DM are complex and multifactorial. Focusing only on each gene or polymorphism provides a limited understanding of the same. Hence, we attempted to detect other potential genes related to DM using the online STRING server. The other ten most probable genes were obtained from the network. Among them, six genes belonged to the interleukin family and three were in the front. Four genes were related to the chemokine (C–X–C motif) ligand family. For example, the first related gene is IL-6R, which is the receptor of the IL-6 gene. Qi et al. reported that the IL6R rs8192284 variant was significantly associated with plasma CRP level and could predict diabetes risk [72]. Jiao et al. performed a meta-analysis and suggested that the IL-1B (-511) T-allele polymorphism is associated with a decreased T2DM risk in East Asians [73]. Silva et al. concluded that functional CXCL8 rs4073, rs2227307, and rs2227306 SNPs are relevant genetic factors for T2DM [74]. Trapali et al. indicated that the TNF-α308G/A polymorphism is significantly associated with T2DM susceptibility [75]. In summary, there is a need toexplore these partners of the IL-6 gene and gene–gene interactions in the development and treatment of DM.

Although we performed a comprehensive meta-analysis, this study has several limitations. First, studies from mixed populations and Africans are limited, which leads to missing or insufficient results and may influence the conclusion. Second, one single gene or one polymorphism may not have the power to result in the development of DM, which is a complex process including gene–gene or gene-environment interactions, and further studies should pay close attention to the same. Third, four databases were included, and some valuable studies from other databases or languages could not be identified, which should have an impact on the current conclusions. Finally, most of the studies were selected using the PCR–RFLP technique in current publications, and the authors may apply to duplicate selected samples for the second time at least 10% of the total samples to confirm the genotypes detected by PCR–RFLP, as real-time PCR is a reference method which can verify the genotyping in PCR–RFLP technique to avoid false positives.

## Conclusions

In summary, our meta-analysis provided evidence that the *IL-6 rs1800795* polymorphism was associated with significantly increased T1DM risk in a mixed population. In contrast, a decreased association was found in T2DM susceptibility in Asians. Consequently, further well-designed large-scale studies, particularly those related to gene–gene and gene-environment interactions, are warranted.

## Supplementary Information

**Additional file 1:** PRISMA 2019 checklist.

## Data Availability

All data generated or analyzed in this study are included in this published article.

## Abbreviations

DM: Diabetes mellitus
GWAS: Genome-wide association studies
IL-6: Interleukin-6
SNP: Single nucleotide polymorphism
HB: Hospital-based
PB: Population-based
SOC: Source of control
PCR–RFLP: Polymerase chain reaction followed by restriction fragment length polymorphism
PCR-SSP: Polymerase chain reaction followed with sequence specific primers
MALDI-TOF: A chip-based matrix-assisted laser-desorption/ionization time-of-flight
